# Treatment Intensification in HIV-Infected Patients Is Associated With Reduced Frequencies of Regulatory T Cells

**DOI:** 10.3389/fimmu.2018.00811

**Published:** 2018-04-30

**Authors:** Eva M. Grützner, Tanja Hoffmann, Eva Wolf, Elke Gersbacher, Ashley Neizert, Renate Stirner, Ramona Pauli, Albrecht Ulmer, Jürgen Brust, Johannes R. Bogner, Hans Jaeger, Rika Draenert

**Affiliations:** ^1^Division of Infectious Diseases, Medizinische Klinik und Poliklinik IV, Ludwig Maximilian University of Munich, Munich, Germany; ^2^German Center for Infection Research, Site Munich LMU, Munich, Germany; ^3^MUC Research GmbH, Munich, Germany; ^4^Dr. Med. Werner Becker, Dr. Med. Ramona Pauli, Gemeinschaftspraxis am Isartor, Munich, Germany; ^5^Dr. Med. Albrecht Ulmer, Dr. Med. Bernhard Frietsch, Dr. Med Markus Müller, Gemeinschaftspraxis, Stuttgart, Germany; ^6^Mannheimer Onkologie Praxis, Mannheim, Germany; ^7^MVZ Karlsplatz, HIV Research and Clinical Care Centre, Munich, Germany

**Keywords:** HIV-1, treatment intensification, immune suppressive cells, myeloid-derived suppressor cells, regulatory T cells, regulatory B cells, CD8 T cell response, New Era study

## Abstract

In untreated HIV infection, the efficacy of T cell responses decreases over the disease course, resulting in disease progression. The reasons for this development are not completely understood. However, immunosuppressive cells are supposedly crucially involved. Treatment strategies to avoid the induction of these cells preserve immune functions and are therefore the object of intense research efforts. In this study, we assessed the effect of treatment intensification [=5-drug antiretroviral therapy (ART)] on the development of suppressive cell subsets. The New Era (NE) study recruited patients with primary HIV infection (PHI) or chronically HIV-infected patients with conventional ART (CHI) and applied an intensified 5-drug regimen containing maraviroc and raltegravir for several years. We compared the frequencies of the immune suppressive cells, namely, the myeloid-derived suppressor cells (MDSCs), regulatory B cells (Bregs), and regulatory T cells (Tregs), of the treatment intensification patients to the control groups, especially to the patients with conventional 3-drug ART, and analyzed the Gag/Nef-specific CD8 T cell responses. There were no differences between PHI and CHI in the NE population (*p* > 0.11) for any of the studied cell types. Polymorphonuclear myeloid-derived suppressor cell (PMN-MDSC), monocytic myeloid-derived suppressor cell (M-MDSC), and the Breg frequencies were comparable to those of patients with a 3-drug ART. However, the Treg levels were significantly lower in the NE patients than those in 3ART-treated individuals and other control groups (*p* ≤ 0.0033). The Gag/Nef-specific CD8 T cell response was broader (*p* = 0.0134) with a higher magnitude (*p* = 0.026) in the NE population than that in the patients with conventional ART. However, we did not find a correlation between the frequency of the immune suppressive cells and the interferon-gamma^+^ CD8 T cell response. In the treatment intensification subjects, the frequencies of the immune suppressive cells were comparable or lower than those of the conventional ART-treated subjects, with surprisingly broad HIV-specific CD8 T cell responses, suggesting a preservation of immune function with the applied treatment regimen. Interestingly, these effects were seen in both treatment intensification subpopulations and were not attributed to the start of treatment in primary infection.

## Introduction

Since 2010, the number of HIV-infected people with access to highly active antiretroviral therapy (ART) increased from 7.5 to 18.2 million in 2016. However, with 36 million people living globally with HIV, only half of them are treated despite intense efforts toward access to ART (1). Thus, there is still an urgent need for alternative treatment strategies with the aim of either eradicating the virus or achieving the so-called “functional cure,” meaning a state of complete suppression of the virus in the absence of medication and a preservation of the immune response.

Conventional 3-drug ART suppresses viral replication and results in an undetectable viral load (VL) in plasma, but an eradication of the virus has not been achieved so far. Within the New Era (NE) study, treatment intensification by adding raltegravir and maraviroc to a 3-drug regimen has been investigated with the goal of eradication or at least a functional cure. The study recruited patients with primary HIV infection (PHI) or patients with chronic HIV infection (CHI). PHI was defined by less than two bands in a Western blot analysis. CHI patients were already treated successfully for at least 36 months with a protease inhibitor (PI) based conventional ART. All the patients had a CD4 nadir above 200 cells/μl and no evidence of resistance to the PI-based regimen. The aim of the study was to stop residual viremia, as well as to reduce and limit the viral reservoir measured by proviral DNA (2–4). Before a possible treatment interruption, correlates of immune control were assessed in all the patients. In earlier studies, a comparable 5-drug ART (also called treatment intensification) was tested with the result that there was no difference with regard to virus suppression or immune activation between the treatment intensification and conventional ART groups (5, 6). However, none of the previous studies evaluated the immune suppressive cells in their cohorts. Therefore, in this study, we hypothesized that an intensified treatment will have a beneficial effect on the development of immune suppressive cells in comparison to conventional 3-drug ART.

In early untreated HIV infection, a strong cytotoxic CD8 T lymphocyte response (CTL)—among others—effectively decreases VL (7–11). However, only <1% of the infected population, namely, elite controllers (EC), reaches complete viral suppression without ART (12–15). One reason for the viral persistence and eventual disease progression is the loss of CTL function over the disease course (e.g., production of cytokines/chemokines, degranulation) (16, 17). This phenomenon is termed immune exhaustion. In addition to immune checkpoints, e.g., PD-1 and CTLA-4, immune suppressive cells are said to be responsible for immune exhaustion. In HIV infection, well-known immune suppressive cells are regulatory T cells (Tregs). More recently, myeloid-derived suppressor cells (MDSCs) and regulatory B cells (Bregs) have been described to inhibit T lymphocyte function as well, leading to the loss of immune control in chronic viral infections.

In HIV infection, the role of Tregs is ambiguous and has not been evaluated conclusively (18–25). On the one hand, Tregs were found to reduce immune activation, which is a positive effect in uncontrolled HIV infection. On the other hand, these cells inhibit T cell responses, leading to viral persistence (18, 20, 23).

MDSCs are immature cells with a myeloid origin, which act immunosuppressively by inhibiting the functions of T lymphocytes, among others. MDSCs are divided into two major subsets, including polymorphonuclear (PMN) and monocytic (M) MDSCs (26, 27). In natural HIV infection, PMN-MDSC as well as M-MDSC frequencies correlate with the VL and are inversely related to the CD4 cell count. ART decreases PMN-MDSC levels within 6 weeks (28, 29). However, data on SIV-infected monkeys report a minor percentage of M-MDSCs within the complete MDSC population and higher levels of PMN-MDSCs during ART than that before infection (30).

Recently, studies by Siewe et al. described a direct correlation of Breg frequencies and CD8 T cell exhaustion in HIV-infected patients (31). Interleukin (IL)-10^+^ Breg frequencies are significantly elevated in viremic patients compared with HIV-uninfected individuals (32, 33). However, even ART-treated patients and EC still have elevated Breg levels when compared with uninfected controls (33).

With the NE study, we had the opportunity to evaluate the impact of treatment intensification on the described immunosuppressive cells in correlation with HIV-specific CD8 T cell responses. We hypothesized that these patients, especially the PHI group, have preserved immune functions and, therefore, low levels of Tregs, MDSCs, and Bregs in comparison to patients treated with conventional 3-drug ART.

## Materials and Methods

### Study Subjects

A total of 116 individuals participated in the study after signing an informed consent form.

New Era: 24 patients within the scope of an amendment of the NE study [EudraCT Number 2008-002070-35, approved by the Bayerische Landesärztekammer (BLAEK) and the German federal institute for drugs and medical devices (BfARM)] and 92 individuals within 4 control groups (study approval by the Institutional Review Board of the Ludwig-Maximilians-Universität, Munich, Germany). The treatment intensification within the NE study consisted of two nucleoside reverse transcriptase inhibitors, a PI, and maraviroc and raltegravir in all cases. Patients with a virus with CXCR4 tropism were excluded from the study.

Primary HIV infection (=subgroup of NE patients with PHI, which was defined by less than two bands in a Western blot analysis; four patients were recruited in Fiebig stage 3 and six patients in Fiebig stage 4; *n* = 10).

Chronic HIV infection (=subgroup of NE patients with CHI; the patients were successfully treated with PI-based ART for at least 36 months; *n* = 14).

At time of immunological analysis, the median duration of intensified treatment (=5 drugs) of all the NE patients was 5.5 years. Thus, CHI had a median ART duration of 11/9.5–16.5 years (median/range).

Control groups are defined as follows:

Progressors (PR) (untreated patients in CHI; *n* = 27): CD4 cell count below 400/μl, VL above 10,000 copies/ml.
Controllers (CO) (patients who control HIV infection spontaneously in the absence of ART for at least 3 years; *n* = 21): CD4 cell count above 500/μl and VL below 2,000 copies/ml (including EC: patients with VL below 40 copies/ml; *n* = 8).
3ART (patients treated with a conventional 3-drug ART for at least 4 years; *n* = 21): any CD4 cell count and VL below 50 copies/ml. The median duration of treatment: 10.5/4–18.5 years (median/range). One individual in this group was treated with maraviroc, but no one was treated with raltegravir. No threshold was set for the CD4 count, because this was not done in any of the treated groups.
HIV-uninfected controls (HC) (*n* = 23).

We included two exceptions of those definitions: PR 22 had a low CD4 cell count of 80 cells/μl and, therefore, we accepted a low-level viremia of 2,100 copies/ml. Whereas CO 13 was an EC (infected for 29 years) with a VL below 40 copies/ml and a CD4 count of 467 cells/μl in the absence of ART.

None of the ART-treated patients experienced treatment failure.

To further interpret our data, we stratified the cohort in the following subgroups:

All the patients on any treatment regimen were summarized in the subgroup with ART (w ART) and in analogous patients untreated in the subgroup without ART (w/o ART).

The clinical characteristics of the study subjects are shown in Table 1.

**Table 1:** Characteristics of the study subjects.

	NE (*n* = 24)	PHI (*n* = 10)	CHI (*n* = 14)	3ART (*n* = 21)	PR (*n* = 27)	CO (*n* = 21)	HC (*n* = 23)
Gender	m = 20/f = 4	m = 9/f = 1	m = 11/f = 3	m = 18/f = 3	m = 21/f = 6	m = 13/f = 8	m = 9/f = 14

Age at blood draw (years)	48 (24–62)	47.5 (24–56)	49.5 (36–62)	49 (30–71)	40 (20–60)	39 (23–66)	45 (25–62)

CD4 pre-ART (abs., %)	434 (212–751) 16.5% (7–37)	467 (339–751) 23.5% (7–37)	247 (212–489) 13.5% (8–23)	294 (12–492) 19% (1–35)	n.a.	n.a.	n.a.

CD4 at blood draw (abs., %)	713 (551–1,342) 37% (19–57)	892 (557–1,342) 42.5% (37–57)	676 (551–1,052) 31% (19–50)	604 (104–1,243) 31% (9–56)	136 (25–357) 11.5% (1–26)	706 (467–1,442) 35% (16–48)	n.a.

CD4 nadir (abs.)	360 (212–751)	467 (339–751)	237 (212–530)	235 (6–472)	143 (12–357)	534 (270–1,259)	n.a.

VL pre-ART (cp/ml)	299,230 (6,769–39,791,860)	1,410,751 (33,873–39,791,860)	173,642 (6,769–501,187)	40,749 (2,583–758,578)	n.a.	n.a.	n.a.

VL at blood draw (cp/ml)	<40 (<40–150)	<40 (<40–150)	<40 (<40)	<40 (<40)	123,818 (2,100–2,970,000)	103 (<40–1,214)	n.a.

Treatment length (years)	5.5 (4.5–6.5) (=only intensified ART)	6 (5–6.5)	5.5 (4.5–6.5) Total years of ART: 11 (9.5–16.5)	10.5 (4–18.5)	n.a.	n.a.	n.a.

*NE, patients in the New Era study; PHI, subgroup of the NE patients with primary HIV infection (4 patients of Fiebig stage 3; 6 patients of Fiebig stage 4); CHI, subgroup of the NE patients with chronic HIV infection; HC, HIV-uninfected controls; CO, controllers; PR, progressors; 3ART, patients treated with a conventional 3-drug ART; m, male; f, female; n.a., not applicable; abs., absolute; ART, antiretroviral therapy*.

*The data are presented as the median and range*.

*For the subgroup PHI: the CD4 count pre-ART and CD4 nadir are the same, since there was only one blood draw after the diagnosis of HIV infection and before the start of ART*.

*For viral load: the limit of detection was 40 cp/ml in this study*.

*The CD4 counts between the NE and 3ART patients at blood draw were significantly different (*p* = 0.044; Mann–Whitney *U* test). However, there was no difference in the CD4 count at blood draw between the CHI and 3ART patients (*p* = 0.288, Mann–Whitney *U* test). Therefore, the difference was driven by the PHI patients who had higher CD4 counts*.

### Peripheral Blood Mononuclear Cell (PBMC) Isolation

Peripheral blood mononuclear cells were isolated from freshly obtained EDTA blood by Ficoll density gradient centrifugation (Biocoll Separation Solution, Biochrom, Germany). The frequencies of the PMN-MDSCs and M-MDSCs (including isotype control) were determined in fresh PBMCs directly after blood draw (≤2 h after blood draw). For this reason, only individuals living in Munich were included in the MDSC studies. The frequencies of the Bregs and Tregs as well as the IL-10 production by the Bregs and the T cell immune responses by interferon-gamma enzyme-linked immunospot (Elispot) assay were determined using frozen PBMCs from all the study subjects.

### Flow Cytometric Analysis

The extracellular staining was performed with fluorescent antibodies using fresh (PMN-MDSCs/M-MDSCs) or frozen (Tregs/Bregs) PBMCs as previously described (28). The following antibodies were used: PMN-MDSCs: CD11b-FITC, CD14-APC, CD15-PerCP, and CD66b-PE; M-MDSCs: CD11b-FITC, CD14-APC, CD33-PE, HLA-DR-PerCP, and PerCP isotype control; Bregs: CD19-PerCP, CD24-APC, and CD38-FITC; and Tregs: CD4-APC and CD25-FITC (all BioLegend, USA). IL-10 production in the Bregs was determined with an intracellular cytokine staining protocol for IL-10-PE (BioLegend). The Tregs were stained intracellularly with anti-FoxP3-PE (BioLegend) using the FoxP3 staining buffer set (eBiosciences, USA) [as described in Ref. (21)]. The cells were analyzed on a FACSCalibur (BD, Germany), and the data analysis was done using FlowJo software 7.2.1 (TreeStar, Inc., Ashland, OR, USA). The gating strategies were according to Vollbrecht et al. (28) and Rieber et al. (34, 35) for PMN-MDSCs. For the gating of the M-MDSCs, we used a PerCP isotype control to gate for HLA-DR and our gating strategy was according to Dumitru et al. (36). Bregs were gated as CD19^+^, CD24^hi^, and CD38^hi^ (31, 33), and the Tregs were CD4^+^, C25^+^, and FoxP3^+^. For the gating strategies of all the cell types, see Figure S1 in Supplementary Material. For the statistics, we indicated the MDSCs, Bregs, and Tregs as the percentage of PBMCs in all the subgroups because the number of monocytes vary substantially in the subjects with chronic viral infections. Each patient was tested once for flow cytometric analysis.

### Functional Analysis of the Bregs

The Bregs show an immunosuppressive function by the production of IL-10. For measuring IL-10 production, we stimulated the thawed PBMCs of five PR and five HC in two ways [according to Siewe et al. (31, 33)]. First, the cells (10^6^ PBMCs/ml) were incubated for 48 h with 10 µg/ml CPG-B (oligodeoxynucleotide-2006), 2 µg/ml PAM (palmitoyl-3-cysteine-serine-lysine-4), and 2 µg/ml CD40L (all InvivoGen, San Diego, CA, USA) at 37°C and in 5.0% CO_2_, and during the last 5 h, the cells were supplemented with 1 µg/ml ionomycin, 50 ng/ml phorbol-12-myristate-13-acetate (PMA), 50 µg/ml brefeldin A (all Sigma), and 1 µl/ml monensin (BD Golgi Stop™ BD Biosciences). Second, we only stimulated the cells with ionomycin/PMA. After 2 h of incubation at 37°C/5.0% CO_2_, brefeldin A and monensin were added, and the cells were incubated for another 5 h. After the incubation, the cells were washed and stained intracellularly for IL-10. The background range was <0.02% for IL-10 production.

### Peptides

Overlapping synthetic peptides corresponding to the HIV proteins Gag and Nef were used for screening (15–20 amino acids long, overlap of 5–10 amino acids; Gag: HIV-1 SF-2, Nef: HIV-1 Bru, NIBSC, England). The peptides had a purity of ≥70%.

### Interferon-Gamma Elispot

HIV-specific CD8 T cell responses were quantified by an interferon-gamma Elispot assay using frozen PBMCs (0.5–1 × 10^5^/well) and peptides (final concentration: 12.5 µg/ml) as described previously (37, 38). Interferon-gamma producing cells were counted by direct visualization on an AID Elispot Reader (Autoimmun Diagnostika GmbH, Strassberg, Germany) and are expressed as spot-forming cells (SFC)/10^6^ PBMCs. According to Addo et al., the wells were counted as positive if they had at least the total number of the three control values and were >50 SFC/10^6^ PBMCs (39). The mean of the control values was subtracted of SFC/10^5^. As the upper cutoff limit, 2,000 SFC/10^6^ PBMCs was chosen. The breadth of the CD8 T cell responses was also determined according to Addo et al. (39): responses to two neighboring overlapping peptides were counted as one response toward one epitopic region, since some T cell epitopes are located in the overlap of the two peptides. In addition, for the total magnitude of the CD8 T cell response, we only considered the higher response of the two neighboring overlapping peptides. This conservative approach may potentially underestimate the real number or the real magnitude of the CD8 T cell responses.

### Statistical Analysis

The statistical analyses were performed using GraphPad Prism version 5.0. In most cases, our data were not normally distributed. Therefore, we only used non-parametric tests. Comparisons between two groups were done with the Mann–Whitney *U* test, and comparisons between more than two groups were first tested with the Kruskal–Wallis test. If this was significant (*p* < 0.05), we also did pairwise Mann–Whitney *U* tests with Bonferroni Correction for multiple testing. Four pairwise comparisons were considered relevant and were tested for each experiment as follows: NE vs. 3ART; NE vs. HC; NE vs. PR; and NE vs. CO. The corrected level of significance, therefore, was *p* < 0.0125 when HC was included, and it was *p* < 0.0167 in the experiments without HC. Spearman rank test was used for correlation analyses and Wilcoxon signed rank test for paired comparisons (level of significance *p* < 0.05). Only tests with significant results are indicated in the figures.

## Results

### Comparable PMN-MDSC Frequencies in NE (=Treatment Intensification) and 3ART Patients

**Figure 1:**
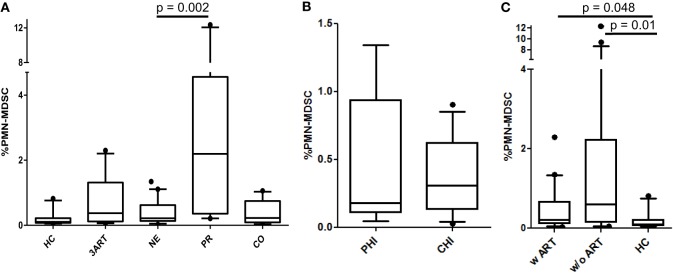
Frequencies of polymorphonuclear myeloid-derived suppressor cells (PMN-MDSCs). **(A)** New Era (NE) showed comparable frequencies to 3ART, but significantly lower frequencies than progressors (PR) (*p* = 0.002). **(B)** Comparable frequencies in the subgroups primary HIV infection (PHI) and chronic HIV infection (CHI) (*p* = 0.97). **(C)** The PMN-MDSC levels of patients with antiretroviral therapy (w ART) and w/o ART were significantly increased compared with the HIV-uninfected controls (HC) group (*p* < 0.048) [HC: *n* = 10; 3ART: *n* = 10; NE: *n* = 19 (PHI: *n* = 8; CHI: *n* = 11); PR: *n* = 10; CO: *n* = 10].

To evaluate the impact of the intensified ART regimen in NE patients on the frequencies of PMN-MDSCs, the levels were compared with 3ART patients and to the HC, CO, and PR patients. We observed significantly lower PMN-MDSC frequencies in NE vs. PR patients (*p* = 0.002). However, there was no difference to the 3ART group (*p* = 0.65) (Figure 1A). The treatment intensification subgroups PHI and CHI had comparable PMN-MDSC frequencies (*p* = 0.97) (Figure 1B). We further stratified all the patients with any ART regimen (w ART: NE and 3ART) and patients without therapy (w/o ART). Both groups had significantly higher percentages of PMN-MDSCs vs. the HC group (w ART vs. HC: *p* = 0.048; w/o ART vs. HC: *p* = 0.01) (Figure 1C).

Thus, our analysis shows low PMN-MDSC frequencies in the treatment intensification-treated individuals, which were, however, comparable to the 3ART patients. In addition, all the ART-treated subjects had PMN-MDSC levels that did not reach the level of the HIV-uninfected controls.

### Comparable M-MDSC Frequencies in All the HIV-Infected Groups

**Figure 2:**
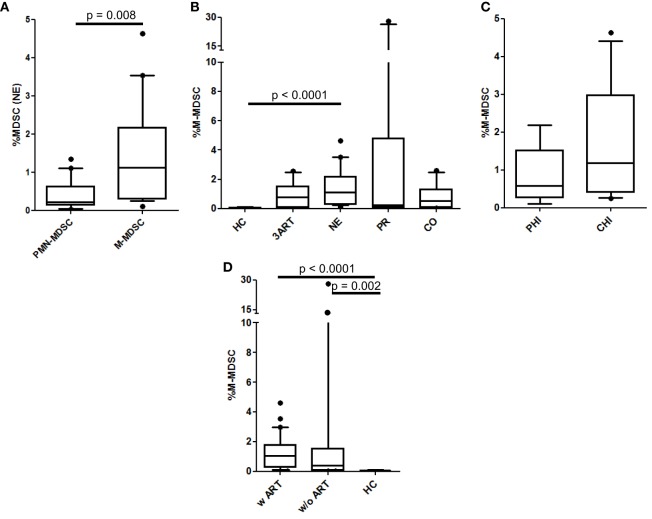
Monocytic myeloid-derived suppressor cell (M-MDSC) frequencies. **(A)** Polymorphonuclear myeloid-derived suppressor cell (PMN-MDSC) levels were significantly lower than M-MDSCs in the New Era (NE) population (*p* = 0.008). **(B)** M-MDSCs in the NE group were similar to the 3ART patients. **(C)** Comparable frequencies in the subgroups primary HIV infection (PHI) and chronic HIV infection (CHI) (*p* = 0.2). **(D)** The M-MDSC levels in the patients with antiretroviral therapy (w ART) and w/o ART were significantly increased compared with the HIV-uninfected controls (HC) group (*p* < 0.002) [HC: *n* = 10; 3ART: *n* = 10; NE: *n* = 19 (PHI: *n* = 8; CHI: *n* = 11); progressors (PR): *n* = 10; CO: *n* = 10].

In HIV infection, M-MDSCs are suggested to play a role in T lymphocyte suppression (29, 40). Interestingly, in the NE patients, the frequencies of the M-MDSCs were significantly higher than the PMN-MDSCs (*p* = 0.008) (Figure 2A), whereas there was no significant difference between these cells in the PR patients (*p* = 0.65) (data not shown). In contrast to the PMN-MDSCs, the percentages of M-MDSCs in our cohort were significantly higher in the treatment intensification patients than those in the HC patients (*p* < 0.0001) but were comparable to those in the 3ART patients (*p* = 0.21) (Figure 2B). Again, within the NE groups, the PHI and CHI subgroups showed comparable values (*p* = 0.2) (Figure 2C). In accordance with these data, the analyses in patients with or without ART showed comparable frequencies, which were significantly higher than those in the HC group (*p* < 0.002) (Figure 2D).

Taken together, in our study, the M-MDSC levels in treatment intensification patients were not different than the 3ART group and were significantly higher than the PMN-MDSCs.

### Low Breg Frequencies in the Treatment Intensification and the 3ART Groups

**Figure 3:**
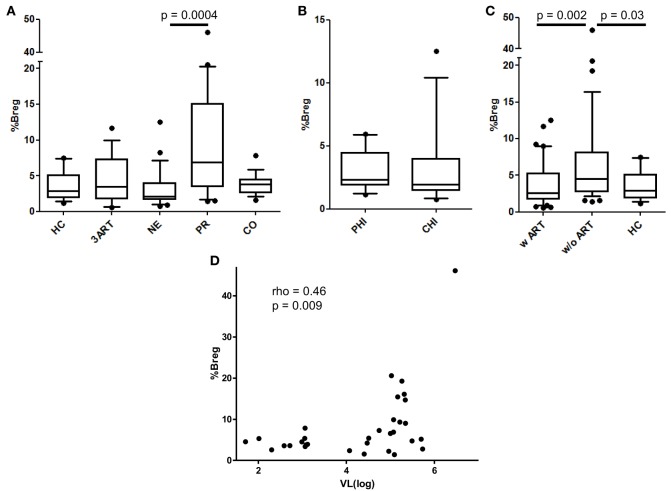
Regulatory B cell (Breg) levels and correlation of their frequencies with viral load (VL). **(A)** There was no difference in the Breg frequencies between the New Era (NE) and 3ART patients, but the NE patients showed significantly lower Breg frequencies than progressors (PR) (*p* = 0.0004). **(B)** Again, there were comparable frequencies in the subgroups primary HIV infection (PHI) and chronic HIV infection (CHI) (*p* = 0.46). **(C)** The Breg levels of the patients with antiretroviral therapy (w ART) were significantly decreased compared with those w/o ART (*p* = 0.002) and were similar to the HIV-uninfected controls (HC) group. **(D)** The Breg frequencies directly and significantly correlated with the VL of the viremic HIV-infected patients of our cohort (rho = 0.46, *p* = 0.009) [HC: *n* = 16; 3ART: *n* = 16; NE: *n* = 24 (PHI: *n* = 10; CHI: *n* = 14); PR: *n* = 21; CO: *n* = 17].

Since Bregs are a group of immunosuppressive cells only recently described in HIV infection, we evaluated them in our cohort (31–33). According to Siewe et al., we defined the Bregs as CD19^+^, CD24^hi^, and CD38^hi^ and showed IL-10 production in these cells for a subset of the PR and CO patients. We found significantly increased IL-10 production in stimulated vs. unstimulated Bregs in all 10 samples tested independent of the stimulation type [for ionomycin *p* = 0.002, for toll-like receptor (TLR) agonists *p* = 0.002; Figure S2 in Supplementary Material]. Overall these results showed that, in our assays, phenotypically defined Bregs are capable of IL-10 production, and therefore, they are Bregs. As previously described, we observed a positive correlation of Breg frequencies with VL in viremic HIV-infected patients (rho = 0.46, *p* = 0.009) (Figure 3D).

The NE patients had comparable levels of Bregs compared with those of the 3ART patients and the HC group (Figure 3A). Their Breg frequencies were significantly lower than those of the untreated group of PR patients (*p* = 0.0004) (Figure 3A). The PHI and CHI subgroups showed no difference in Breg frequencies among each other (*p* = 0.46) (Figure 3B). Stratifying the patients in the w ART and w/o ART groups revealed that the w ART group showed significantly lower frequencies than those of the w/o ART group (*p* = 0.002), and these frequencies were comparable to those of the HC group (*p* = 0.61) (Figure 3C).

In conclusion, Breg levels in the NE patients were not different than those of the 3ART patients.

### Significantly Lower Relative Treg Frequencies in the NE Patients Compared With 3ART Subjects

**Figure 4:**
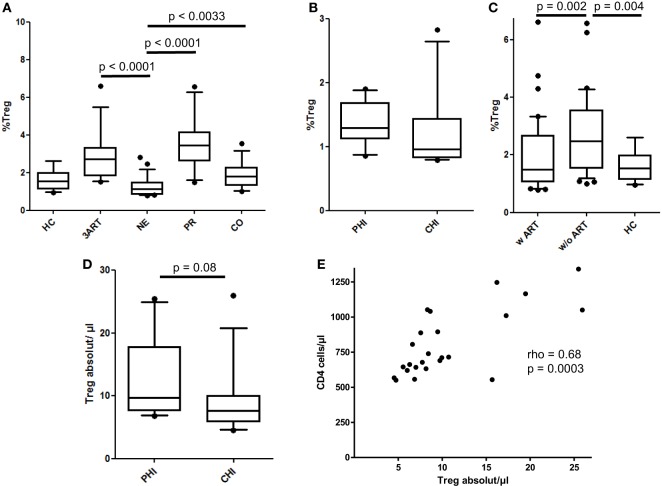
Relative frequencies and absolute cell counts of regulatory T cells (Tregs). **(A)** The New Era (NE) group showed the significantly lowest Treg frequencies compared with all the other HIV-infected groups (*p* < 0.0033). **(B)** Comparable frequencies in the subgroups primary HIV infection (PHI) and chronic HIV infection (CHI) (*p* = 0.11). **(C)** As already shown in the regulatory B cells, the relative Treg levels in the patients with antiretroviral therapy (w ART) were significantly decreased compared with those w/o ART (*p* = 0.002) and were similar to the HIV-uninfected controls (HC) group. **(D)** There was a trend of a higher absolute Treg number in the PHI subgroup than in CHI subgroup (*p* = 0.08). **(E)** The absolute Treg cell count in the NE group significantly correlated with the CD4 cell counts (rho = 0.68, *p* = 0.0003) [HC: *n* = 14; 3ART: *n* = 15; NE: *n* = 24 (PHI: *n* = 10; CHI: *n* = 14); progressors (PR): *n* = 18; CO: *n* = 17].

As a subset of CD4 T cells, Treg frequencies vary during the course of HIV infection, when CD4 T cells diminish with disease progression. Thus, the differentiation between the relative frequencies and the absolute numbers of Tregs is important to compare our results to those of other studies. In the NE group, the relative Treg levels were highly significantly lower compared with those of the 3ART patients (*p* < 0.0001) (Figure 4A). They were also lower than those in the PR and CO groups (*p* < 0.0001 and *p* = 0.0033, respectively), whereas the difference compared with the HC group failed to reach statistical significance (Figure 4A). The PHI and CHI subgroups were again similar (*p* = 0.11) (Figure 4B). The entity of patients with ART had lower frequencies of relative Tregs than those of the control groups without ART (*p* = 0.002) (Figure 4C).

In absolute cell numbers, the NE subgroups and the total NE population had markedly higher Tregs than those of the PR group (*p* = 0.0004, PHI; *p* = 0.02, CHI; *p* = 0.0006, NE) (data not shown). A higher median CD4 cell count in the PHI subgroup than that in CHI (median = 875 cells/μl and median = 676 cells/μl, respectively, however, not statistically significant *p* = 0.107) along with the abovementioned comparable Treg frequencies resulted in higher absolute Treg cell counts in the PHI groups compared with those in the CHI group, which just missed statistical significance (*p* = 0.08) (Figure 4D). In addition, the NE patients showed a significantly strong correlation in the absolute numbers of Tregs with the CD4 cell counts (rho = 0.68, *p* = 0.0003) (Figure 4E).

Taken together, the relative Treg levels were significantly lower in the NE patients than those in the 3ART patients. For this cell type, treatment intensification seems to be advantageous compared with conventional ART.

### No Correlation Between the Levels of Immune Suppressive Cells and the Gag/Nef-Specific CD8 T Cell Response in Treatment Intensification

Immune suppressive cells inhibit CD8 T cell responses. Therefore, we evaluated if there was a correlation between the HIV-specific CD8 T cell responses and the levels of immune suppressive cells studied here for the patients treated with a 5-drug ART. However, we were not able to demonstrate any correlation between the breadth or magnitude of interferon-gamma^+^ CD8 T cells toward Gag and Nef and the inhibitory cells, namely, the PMN-MDSCs, M-MDSCs, Bregs, and Tregs (data not shown).

A direct association between the levels of inhibitory immune cells and the interferon-gamma positive CD8 T cell response was not found in the treatment intensification.

### Stronger Immune Responses Toward Gag p17 and Gag p15 in the NE Patients Compared With the 3ART Patients

**Figure 5:**
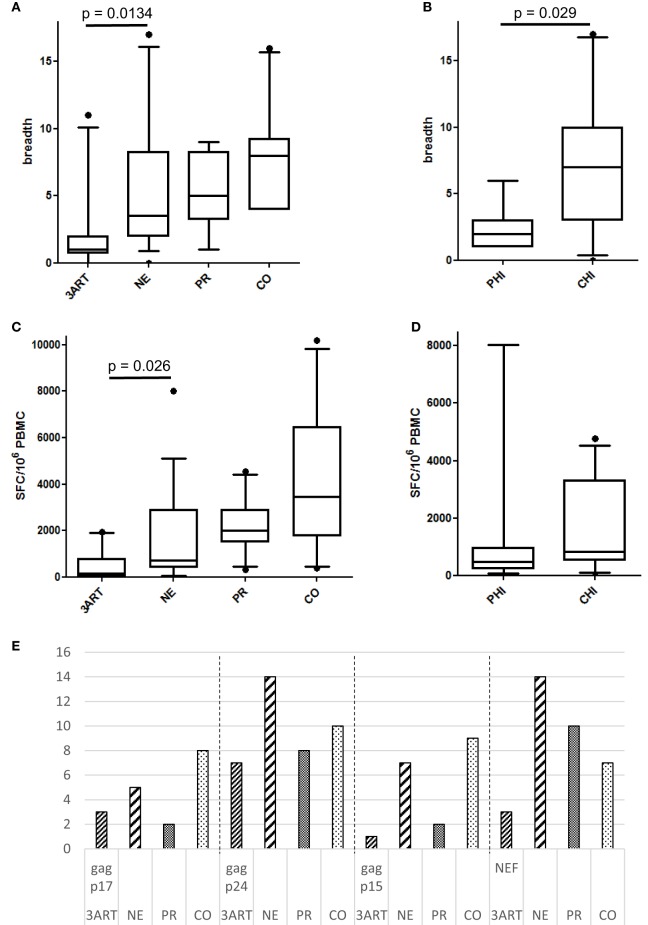
**(A)** Breadth of immune responses in the New Era (NE) and control groups. The NE patients had significantly more CD8 T cell responses in Gag and Nef than the 3ART patients (*p* = 0.0134). **(B)** The chronic HIV infection (CHI) subgroup showed a significantly higher breadth of immune responses than the primary HIV infection (PHI) subgroup (*p* = 0.029). **(C)** The magnitude of the NE group was significantly higher than in the 3ART patients (*p* = 0.026). **(D)** There was no difference between the PHI and CHI subgroups, with regard to the magnitude of the CD8 T cell response (*p* = 0.28). **(E)** Gag p17 and Gag p15 were more often recognized by the NE (especially CHI) and CO groups than by the progressor (PR) and 3ART groups. Spot-forming cells (SFC)/10^6^ peripheral blood mononuclear cells (PBMCs). For the magnitude of the CD8 T cell response, all the individual CD8 T cell responses within Gag and Nef were added for each patient [3ART: *n* = 10; NE: *n* = 18 (PHI: *n* = 7; CHI: *n* = 11); PR: *n* = 10; CO: *n* = 10].

Interestingly, the NE patients revealed a higher number of CD8 T cell responses in Gag and Nef than that of the 3ART patients (*p* = 0.0134) (Figure 5A). In contrast to the comparable frequencies of immunosuppressive cells between the PHI and CHI groups, the breadth of the CD8 T cell responses in the PHI subgroup (median 2, range 1–6) was significantly lower than that in the CHI subgroup (median 7, range 0–17; *p* = 0.029) (Figure 5B), showing that the difference between the NE and 3ART groups was driven by the CHI subgroup. This difference was especially interesting, as both groups—CHI and 3ART—were chronically HIV infected and had comparable times of treatment. In addition, the CD4 counts at blood draw as well as the CD4 counts before the start of ART were comparable in both groups (*p* = 0.288 and *p* = 0.97, respectively). This suggested that it was a very special group that was selected for the CHI group in the NE study.

The total magnitude of the immune responses toward Gag and Nef in the NE patients ranged from 0 to 8,030 SFC/10^6^ PBMCs (median 700) and was therefore significantly higher compared with that of the 3ART patients (*p* = 0.026) (Figure 5C). In contrast to the results in the breadth of the responses, the NE subgroups PHI and CHI showed a comparable magnitude of CD8 T cell responses (*p* = 0.28) (Figure 5D). Thus, the fewer responses in the PHI groups generated stronger amplitudes.

Looking at the distribution of the CD8 T cell responses within the viral proteins, Gag p17 and Gag p15 were more often recognized by the CO group and, interestingly, also by the NE group than by the other groups. Studying this finding in detail showed that the CHI group recognized the Gag subunits p17 and p15, and the PHI group did not. Therefore, the CHI patients were similar to the CO patients in this respect. Gag p24 and Nef were the most frequently recognized protein subunit within all the groups as was to be expected (Figure 5E).

Overall, the CHI subgroup had a higher breadth of CD8 T cell responses compared with that of the 3ART patients and was comparable to the CO patients but did not lead to a higher magnitude of responses than that in the PHI group. There were no significant correlations between the CD8 T cell responses and frequencies of the immune suppressive cells.

## Discussion

The NE study evaluated an intensified antiretroviral treatment strategy with the primary goal of the eradication of HIV-1. We assessed the impact of this 5-drug treatment on several subsets of immune suppressive cells in the NE patients before a possible treatment interruption. Here, we showed that the levels of PMN-MDSCs, M-MDSCs, and Bregs were comparable to those of patients treated with a conventional 3-drug ART. However, the frequencies of the Tregs were significantly lower than those in the 3ART patients, indicating an advantage of the 5-drug regimen.

Interestingly, this effect was seen in both the NE subpopulations (PHI and CHI) and was not attributed to an early start of ART in primary infection. In addition, the NE population showed broader and stronger HIV-specific CD8 T cell responses, mainly driven by the CHI subgroup.

Myeloid-derived suppressor cell levels are significantly elevated in chronic progressive HIV infection. This association is shown for both subsets of MDSCs, namely, PMN-MDSCs (28, 41) and M-MDSCs (29, 42). In SIV infection, PMN-MDSCs increase substantially in acute infection, remain higher than before infection during antiretroviral treatment and rebound after treatment interruption (30). In addition, in human disease, MDSC frequencies decline after the start of ART (28, 29). MDSCs inhibit CD8 and CD4 T cell functions (proliferation, interferon-gamma production) and induce Treg levels (28, 29, 42), thereby contributing to the immune exhaustion in late stage disease. In our cohort, the intensified treatment regimen led to a near-to-normal level of PMN-MDSCs compared with that of the HC subjects, while it was significantly lower than in the PR patients, and there was no difference compared with that of the patients who were treated with a 3-drug ART. As a natural limitation, we do not have PMN-MDSC levels of the time before HIV infection and cannot therefore comment on this comparison. However, within the NE cohort, there are a few patients with elevated PMN-MDSCs to a level we usually do not find in healthy individuals. This might hint to the fact that, also in human disease, PMN-MDSC frequencies do not return to pre-infection levels, as shown in macaques (30)—not even with the start of an intensified treatment regimen in the very early phase of the infection.

The results for the M-MDSCs are not as straightforward. In our study, there was no difference for these cells in any of the HIV-infected groups (including PR), and all the HIV-infected patients had higher M-MDSC levels than those of the HC group. This is in contrast to data published by others (29, 42). One cause for this difference might be environmental or genetic reasons, as these other studies were conducted in dissimilar circumstances. With regard to the data from this study, we cannot postulate a beneficial effect of the 5-drug regimen on M-MDSC levels.

Regulatory B cells were defined just recently as an immune inhibitory cell type in HIV infection and were therefore included in our analysis. They are best characterized by their capability to produce IL-10. Similar to a study by Siewe et al., we defined Bregs by phenotype (CD19^+^CD24^hi^CD38^hi^) (31, 33). However, we studied IL-10 production in a subset of individuals to show that we were in fact dealing with Bregs. Liu et al. had a different approach and evaluated Breg frequencies solely based on IL-10 production (32). In chronic, untreated HIV infection, Breg levels are significantly higher than those in HC, correlate directly with VL and are inversely related with the CD4 count (31, 32, 43). In addition, Bregs inhibit T cell function (31, 32). Our data are consistent with these findings, as we found a significant correlation between the relative Bregs and VL in our viremic patients. The treatment intensification patients had significantly lower Breg frequencies than the CO and PR patients but levels similar to those of the HC group and 3ART-treated subjects. Breg frequencies were already elevated in early HIV infection and may contribute to establishing a viral set point, as well as to disease progression (32). Thus, with an early start of ART, keeping Breg frequencies in a normal range, especially for patients with acute infection, might be a benefit from this treatment strategy. However, since Breg levels between the NE and 3ART patients were comparable, it is debatable if treatment intensification represents an advantage over conventional ART.

In HIV infection, Tregs are the most described and well-known cells with immunosuppressive capabilities. The ambiguous role of Tregs is discussed in several publications (18, 21–23, 44). Cao et al. argued, in reliance to their results, that Tregs have a role rather in disease progression than in hindering immune activation. They described elevated relative frequencies with disease progression (44). Indeed, we observed similar results. The relative Treg frequencies in the HC and CO groups were comparable (*p* = 0.34) and were significantly lower than those in PR and 3ART patients (*p* < 0.009). Surprisingly, the lowest relative Treg frequencies were found in the NE patients with comparable levels in PHI and CHI. The difference between the NE patients and 3ART patients was especially remarkable (*p* < 0.0001), as the levels for those two groups were rather similar for all other cell types. Therefore, treatment intensification seems to have a beneficial effect for this specific cell type. Another reason for this finding could be the inclusion of maraviroc to intensify the treatment in the NE patients. Two studies analyzed the influence of maraviroc on Treg frequencies with contrary results (45, 46). While one study found a decrease in the relative Treg levels in maraviroc-treated individuals compared with maraviroc-free regimens (45), the other trial concluded that the reduction in viremia was the only reason for the Treg decline (46). While all the NE patients received maraviroc as part of their ART, there was only one individual in the 3ART group in our study with a maraviroc containing regimen. This patient had indeed a Treg frequency that was lower than the 3ART median and among the lowest values measured in this group. A third reason for the low Treg levels might be the intensified treatment itself, with lower levels of proviral DNA (especially in PHI) than usually measured in ART-treated patients. In the NE patients, we not only found low Treg frequencies but the absolute Treg numbers correlated well with the CD4 cell counts. This also reflected the healthy Treg compartment in this subgroup.

There was not a significant difference in any of the studied cell types between the PHI and CHI subgroups of the treatment intensification patients, which was surprising to us. We might have found a difference if our study groups had been larger as the highest values were always found in the group of CHI (with the exception of PMN-MDSCs). However, this finding, as it stands, questions the necessity for treatment start in acute HIV infection with regard to immune suppressive cells. On the other hand, the CHI patients in the NE study were chosen very selectively. The patients had to be virologically suppressed by a PI-based ART for at least 3 years and without a history of virological failure or blip (2, 3). It is possible that our findings emphasize this special selection, which is also reflected in the CD8 T cell response, and those patients would have had a benign disease course no matter which treatment.

In our study, we evaluated the CD8 T cell responses on an epitope level to assess possible correlations between the suppressive cells and the breadth of these responses. A limiting factor for this analysis is that the NE patients were treated for several years, and it is known that CD8 T cell responses decline both in breadth and magnitude after the start of ART with the disappearance of viremia (47). We restricted our analyses toward Gag and Nef, which are two of the most recognized HIV-1 proteins by CD8 T cells (39, 48, 49). Therefore, the results are limited to this part of the HIV genome. In our analyses, we did not find any correlations between the levels of the immune inhibitory cells and the CD8 T cell responses. A broad and strong immune response does not necessarily result in control of viral replication (39). In fact, EC control the HI virus below the detection limit but show less breadth and magnitude of immune response than PR (50) and preferentially target the HIV protein Gag (41, 51). In contrast to the similar frequencies of the analyzed cell subsets in the PHI and CHI subgroups, we found a significant difference between these groups in the breadth of the immune response. CHI had a broader CD8 T cell response despite the fact that PHI had higher CD4 counts than CHI. This outcome is in agreement with data from Altfeld et al. (52) and Addo et al. (39), showing that treatment in early infection results in an immune response that is narrower because the CD8 T cells were exposed to an HIV antigen for a shorter period of time. Consistently, all the HIV-1 proteins analyzed were less recognized in the PHI subgroup than in the CHI subgroup.

Gag p17 and p15 contain more variable regions and, therefore, are often less recognized in HIV-infected individuals (39, 49, 53). The CO group had more responses toward these protein subunits than the other groups. Most interestingly, we also found strong responses toward Gag p17 and p15 in the CHI group; these responses were higher than those in the PR and 3ART groups. Assuming that the CO subjects benefit from responses toward Gag p17 and p15, this again stresses the specially selected patients of the CHI group.

With this study, we comprehensively described several immunosuppressive cell subsets in a unique patient population. However, there were some limitations. First, the correlations might not have reached significance because of the small group sizes. The NE study was a multi-center study with a necessity for overnight shipment of samples for some centers. Because of the fragile nature of MDSCs (54–56), we were only able to include patients from Munich in the MDSC analyses. This did not apply to the same degree for the Bregs and Tregs. Therefore, the groups for those assays were slightly larger. Second, we determined the immunosuppressive cells in the peripheral blood. Tregs are found to be underrepresented in this compartment (21), because the cells traffic to the tissues and lymph nodes. To the best of our knowledge, there are no data on humans for the other cells, at present, about other compartments than blood. Thus, future studies might provide insights if these cells act directly on CD8 T cells in the lymphoid tissue.

Among the investigators of the study, it was highly discussed whether to stop treatment in this study population or not. In addition, if the treatment was stopped, should this be done with an additional intervention (e.g., vaccine or drug) or without. We think that it would be particularly interesting to stop treatment in the CHI group because, according to our results, the patients of this group seem very special as described earlier.

## Ethics Statement

This study was carried out in accordance with the recommendations of the Institutional Review Board of the Ludwig-Maximilians-Universität, Munich, Germany with written informed consent from all subjects. Samples of New Era patients were obtained within the scope of an amendment of the New Era study [EudraCT Number 2008-002070-35, approved by the Bayerische Landesärztekammer (BLAEK) and the German federal institute for drugs and medical devices (BfARM)] with written informed consent from all subjects. All subjects gave written informed consent in accordance with the Declaration of Helsinki. The protocol was approved by the Institutional Review Board of the Ludwig-Maximilians-Universität, Munich, Germany.

## Author Contributions

EMG designed the research, acquired the data, performed the experiments, analyzed the data, performed the statistical analysis, and drafted the manuscript. TH acquired the data, performed the experiments, analyzed the data, and performed the statistical analysis. RS and AN acquired the data and performed the experiments. RP, AU, and JB provided samples and edited the manuscript. EW, EG, and HJ provided samples, contributed to the interpretation of the results, and edited the manuscript. JRB provided samples, interpreted the data, and wrote and edited the manuscript. RD designed and oversaw the research, analyzed and interpreted the data, performed the statistical analysis, wrote the manuscript, and revised the final version of the manuscript. All the authors read and approved the final manuscript.

## Conflict of Interest Statement

The authors declare that the research was conducted in the absence of any commercial or financial relationships that could be construed as a potential conflict of interest.

## Abbreviations

3ART, patients treated with a conventional 3-drug ART; Bregs, regulatory B cells; CHI, chronic HIV infection; CO, controllers; EC, elite controllers; HC, HIV-uninfected controls; M-MDSCs, monocytic myeloid-derived suppressor cells; NE, New Era; PHI, primary HIV infection; PMN-MDSCs, polymorphonuclear myeloid-derived suppressor cells; PR, progressors; Tregs, regulatory T cells.
