# Supplementary material for: Treatment Intensification in HIV-Infected Patients Is Associated With Reduced Frequencies of Regulatory T Cells
Source: Front Immunol. 2018 Apr 30;9:811. doi: 10.3389/fimmu.2018.00811 (PMC5936794; doi:10.3389/fimmu.2018.00811)
Supplement: Supplementary file 1 [file image_1.PDF]

S1: Gating strategies of immunosuppressive cells

PMN-MDSCs

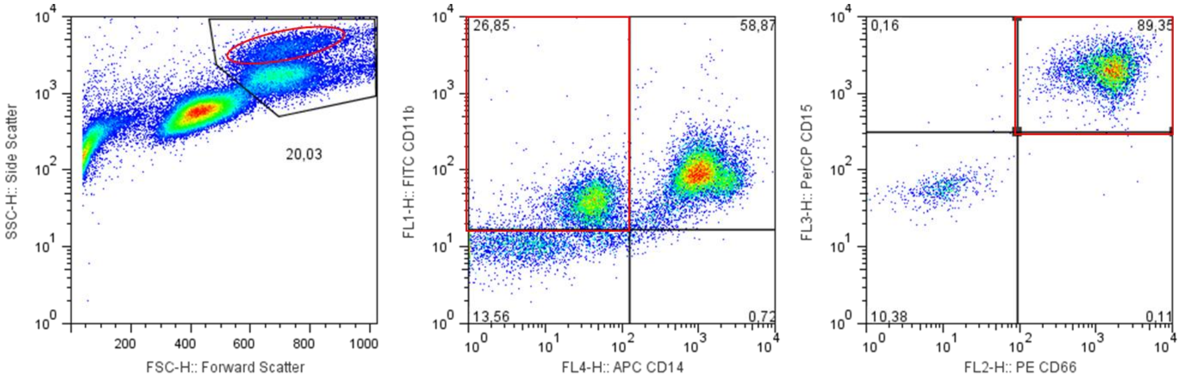

Representative dot blots and gating strategies for PMN-MDSCs. The first gate (black gate, first panel) is placed on the monocyte fraction in FSC and SSC. The main fraction of MDSCs is found in the population above the monocyte fraction (oval red gate, first panel). Second gate shows the $CD11b^{+}$ and $CD14^{-}$ population (red square gate, second panel). In the third panel (red square gate, third panel) cells are $CD11b^{+}/CD14^{-}$ and $CD66b^{+}/CD15^{+}$ and are defined as PMN-MDSC. Gating was performed according to Vollbrecht et al. (AIDS 2012) and Rieber et al. (J Immunol 2013, Clin Exp Immunol 2013).

M-MDSCs

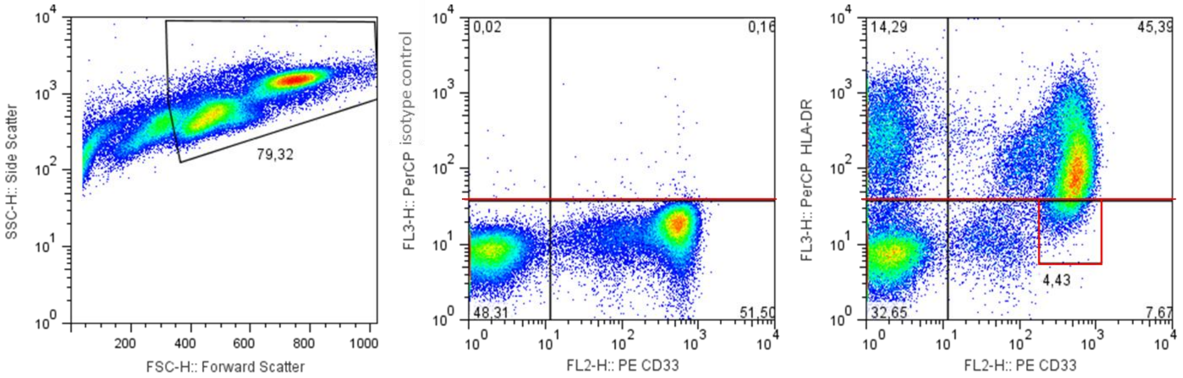

Representative dot blots and gating strategies for M-MDSCs. The first gate (black gate, first panel) is placed on the lymphocyte and monocyte fraction in FSC and SSC. Second panel shows PerCP isotype control (red line, second panel) which defines the HLA-DR negative population. In the third panel (red square gate, third panel) cells are $HLA\text{-}DR^{low/-}$ and $CD33^{+}$ and are defined as M-MDSC. Gating was performed according to Dumitru et al. (Cancer Immunol Immunother 2013).

Bregs

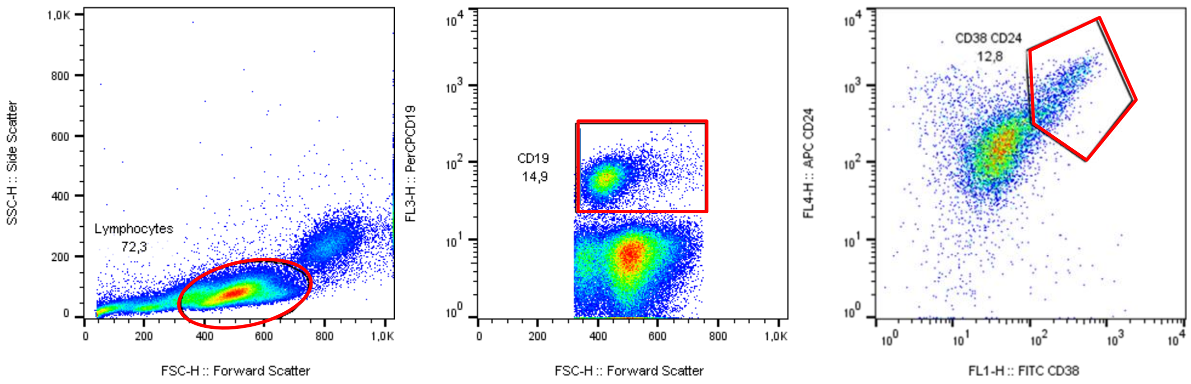

Representative dot blots and gating strategies for Bregs. The first gate (red oval gate, first panel) is placed on the lymphocyte fraction in FSC and SSC. Bregs are defined as CD19^{+} (red gate, second panel). Third gate shows the CD24^{hi} and CD38^{hi} population of CD19^{+} cells (red gate, third panel). Gating was performed according to Siewe et al. (J Leukoc Biol 2013, PLoS One 2014).

Tregs

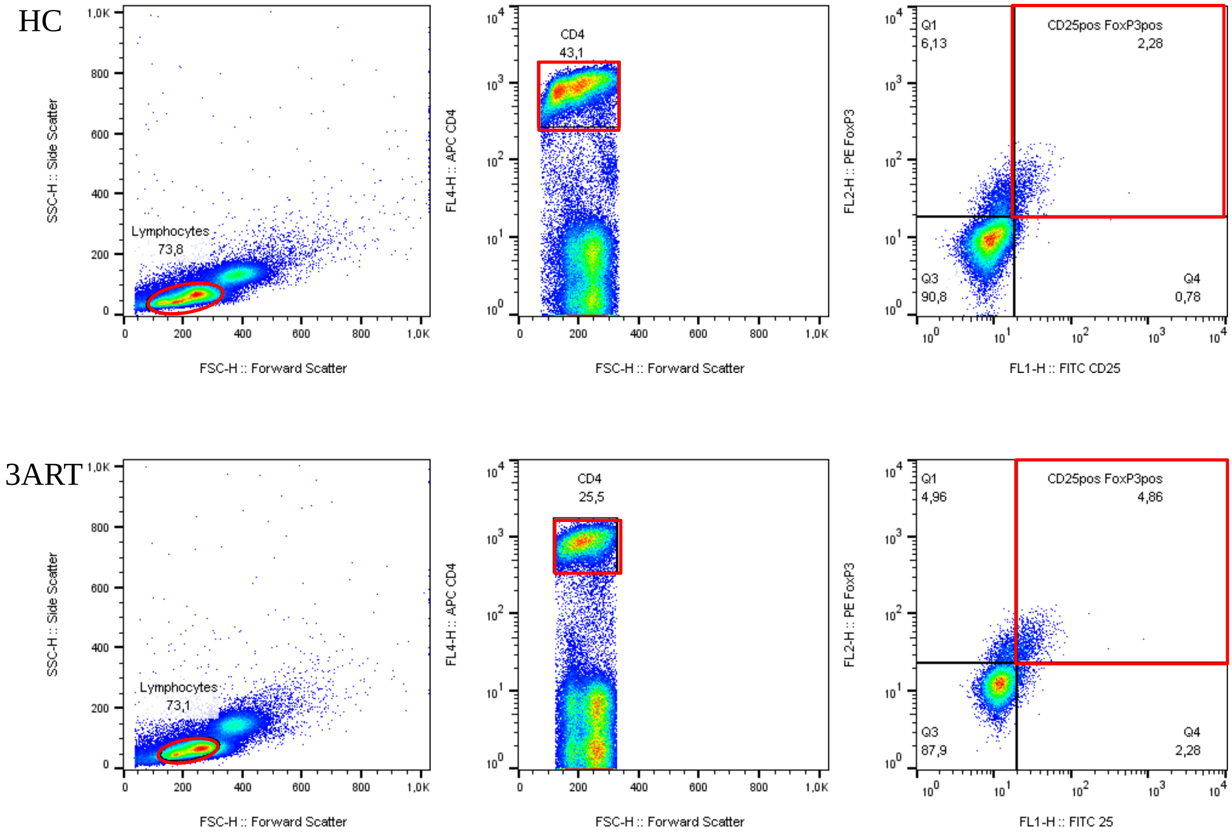

Tregs cont.

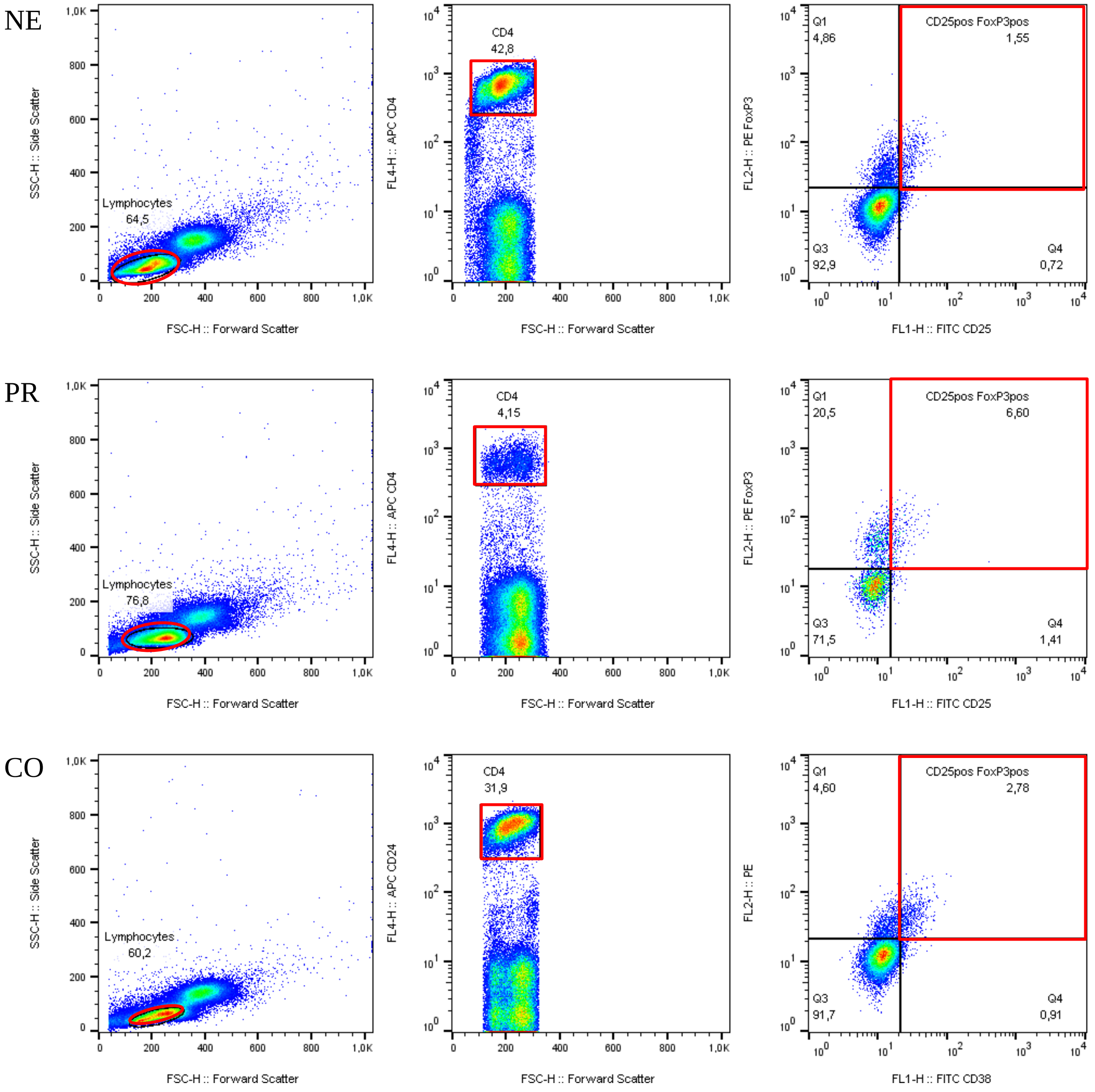

Representative dot blots and gating strategies for Tregs. The first gate (red oval gate, first panel) is placed on the lymphocyte fraction in FSC and SSC. Tregs are defined as CD4$^+$ (red rectangle gate, second panel) and CD25$^+$ and FoxP3$^+$ (red gate, third panel).

S2: Bregs are capable of IL-10 production

A

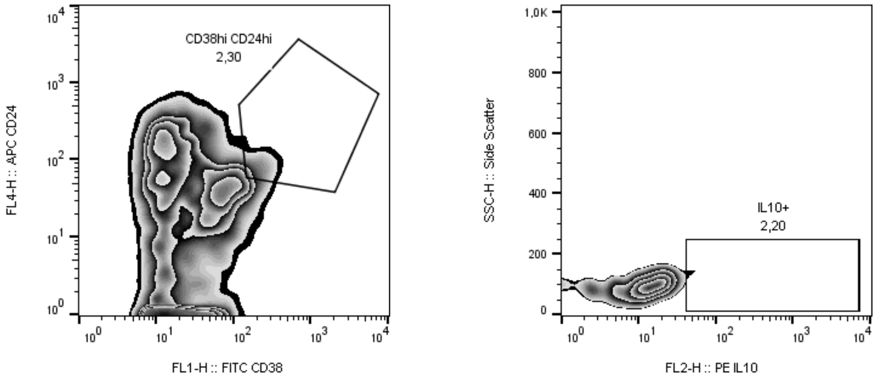

B

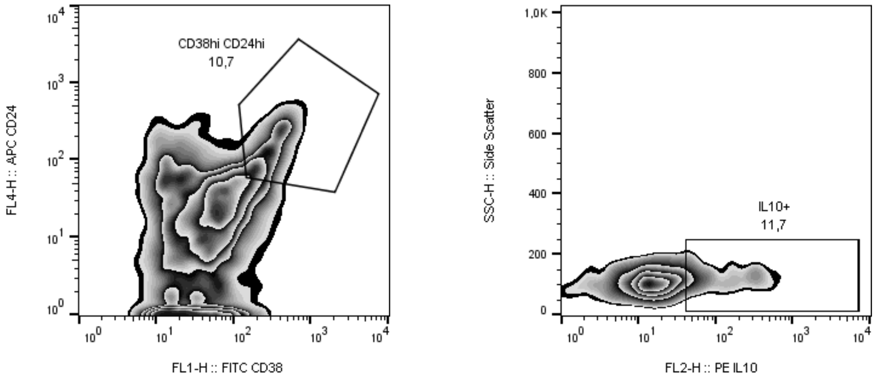

C stimulation type:

TLR agonists Ionomycin

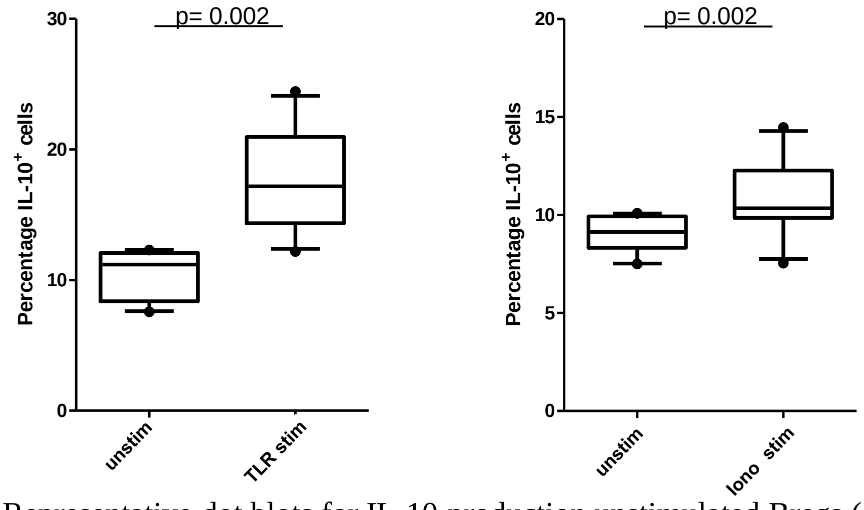

Representative dot blots for IL-10 production unstimulated Bregs (A) and Bregs stimulated with TLR agonists and Ionomycin respectively (B). (A-B) Left panels show $CD19^{+}CD38^{hi}CD24^{hi}$ Bregs (black gate). The right panel of stimulated Bregs (B) shows markedly increased IL-10^{+} cells compared to the unstimulated Bregs (A) of the same HIV infected patient. (C) PBMCs were stimulated with TLR agonists or Ionomycin as described in methods section. Stimulated Bregs showed significantly increased percentages of IL-10^{+} cells compared to unstimulated controls independent of stimulation type ($p= 0.002$; TLR agonists: left panel, Ionomycin: right panel) (C Wilcoxon signed rank test).
